# Responsiveness of hypothalamo-pituitary-adrenal axis to leptin is impaired in diet-induced obese rats

**DOI:** 10.1038/s41387-019-0076-y

**Published:** 2019-03-18

**Authors:** Andrew C. Shin, Sheba M. J. MohanKumar, Priya Balasubramanian, Madhu P. Sirivelu, Katrina Linning, Andrew Woolcock, Michelle James, Puliyur S. MohanKumar

**Affiliations:** 1 Department of Nutritional Sciences, College of Human Sciences, Texas Tech University, Texas, TX 79409 USA; 2 Department of Veterinary Biosciences and Diagnostic Imaging, University of Georgia, Athens, GA 30602 USA; 3 Neuroscience Program, Michigan State University, East Lansing, MI 48824 USA

## Abstract

**Background/objectives:**

Diet-induced obese (DIO) rats have altered stress (HPA) axis activity compared to diet-resistant (DR) rats when chronically exposed to a high-fat (HF) diet. Since stress axis is tightly regulated by leptin, an adipocyte-secreted hormone that is important for controlling body weight, we hypothesized that leptin action is impaired in DIO rats leading to alterations in HPA axis activity.

**Subjects/methods:**

We intraperitoneally injected selectively bred DIO and DR rats with either saline or recombinant rat leptin. HPA axis activity was assessed by measuring norepinephrine (NE) in the paraventricular nucleus (PVN), corticotropin-releasing hormone (CRH) in the median eminence, and serum corticosterone (CORT). To test if HF exposure duration and the corresponding increase in leptin differentially affects HPA axis activity, we placed animals on a chow or HF diet for 1 or 6 weeks.

**Results:**

Leptin injection significantly increased serum leptin levels in both DIO and DR animals. It also reduced PVN NE in both groups, indicating that noradrenergic neurons in both groups remain responsive to leptin. HF diet duration-dependently increased serum leptin only in DIO animals whereas PVN NE increased in both groups. While DR rats responded to HF diet by increasing CRH and CORT at both time-points, responses in DIO rats varied, suggesting that they have altered HPA axis activity that may be dependent on HF-induced leptin levels and/or signaling. To understand the underlying mechanisms, we measured pSTAT-3, a marker of leptin signaling, in brainstem noradrenergic neurons and found reduced pSTAT-3 in A1 region of HF-fed DIO rats. We also found higher serum free fatty acids (FFAs) and a pro-inflammatory cytokine, IL-1β.

**Conclusions:**

Collectively, these findings reveal that DIO rats have inherent neuroendocrine impairment in NE-HPA axis circuitry that worsens with the extent of HF diet exposure, possibly due to brainstem leptin resistance and/or elevated circulating FFAs and IL-1β.

## Introduction

Diet-induced obesity (DIO) is a serious health condition that has been affecting individuals of all ages. Stress is one of many causes that has long been implicated as a contributing factor for the development of obesity^1^. Stress alters brain circuits to favor consumption of palatable, high-energy foods, promotes lipid accumulation in liver, and increases insulin secretion to promote adiposity^2^. Activation of the stress axis involves increased noradrenergic outflow to the paraventricular nucleus of the hypothalamus (PVN) that contains corticotropin-releasing hormone (CRH) neurons. Stimulation of CRH neurons by norepinephrine (NE) results in CRH release in the median eminence (ME) that is transported to the anterior pituitary to stimulate corticotrophs to secrete adrenocorticotropic hormone (ACTH). ACTH then acts on the adrenal cortex to stimulate secretion of corticosterone (CORT). Since stress plays a key role in driving anabolism, we had suspected that high-fat (HF) diet would further stimulate the stress axis, contributing to the perpetuation of a vicious cycle leading to diet-induced obesity. However, our previous work demonstrated that HF feeding for 6 weeks does not increase stress axis activity, but rather induces characteristic dysregulation of the stress axis in DIO rats^3^. Specifically, HF feeding increased NE levels in the PVN of DIO rats, but failed to produce a corresponding increase in CRH and CORT as demonstrated in diet-resistant (DR) rats.

HF diet increases fat mass and hence secretion of leptin from the adipose tissue^4^. Similarly, CORT increases leptin gene expression^5^ and stimulates leptin secretion^6^. Leptin, in turn, works as a negative feedback signal to suppress the stress axis. In support of this concept, studies have shown the ability of leptin to decrease NE release in the PVN (i.e., leptin action) and concurrently decrease serum corticosterone in rats^7^, NE efflux from the hypothalamus^8^, and to suppress ACTH and corticosterone in mice^9^. Although HF feeding for 6 weeks does increase circulating leptin in DIO rats, the persistent increase in NE levels in the PVN suggests leptin insensitivity in NE neurons^3^. Evidence suggests that leptin resistance in neurons is most likely due to impaired downstream signaling that involves phosphorylated signal transducer and activator of transcription-3 (pSTAT-3)^10^ and suppressor of cytokine signaling-3 (SOCS-3)^11^, the negative feedback inhibitor of leptin signaling. Increases in circulating free fatty acids (FFAs), triglycerides, and/or pro-inflammatory cytokines may have independent effects in stimulating hypothalamic NE or inducing leptin insensitivity^12–18^. We hypothesized that leptin resistance in NE neurons (1) is mediated through downstream pSTAT-3 pathway, (2) is progressive with increased duration of HF feeding, and (3) is associated with changes in circulating FFAs and cytokines.

Therefore, in the present study, we treated DIO and DR rats with a single dose of recombinant rat leptin or subjected the rats to HF feeding for 1 or 6 weeks. We followed the changes in different arms of the stress axis and investigated the effects of HF diet exposure on serum FFAs and cytokine levels, as well as pSTAT-3 expression in brainstem noradrenergic neurons to determine the site of leptin insensitivity.

## Methods

### Animals

Breeding pairs of polygenically obese DIO (i.e., obesity-prone) and DR rats were obtained from Charles River Laboratories, Inc. (Wilimington, MA). They were housed in temperature-controlled (23 ± 2 °C) rooms on a 12:12-h light-dark schedule with *ad libitum* access to food and water. They were bred in our colony and offspring were weaned after 1 month. After weaning, the offspring were single-housed and fed standard chow diet (SC; Teklad 8640 diet; 3.11 kcal/g, 5% fat; Harlan, Indianapolis, IN) until they were used in the experiments. Sample size for each group is chosen based on prior studies in our laboratory with similar protocols. Animals were handled every day to minimize any potential stress before and during the experiments. All studies were conducted in accordance with the National Institutes of Health’s *Guide for the Care and Use of Laboratory Animals* and the protocol was approved by the institutional animal care and use committee at Michigan State University.

### Treatment

In first experiment, overnight-fasted 3-month old male DIO and DR rats (6–8 per group) were either injected intraperitoneally with 250 µl of saline or 500 µg of rat recombinant leptin (R&D systems, Minneapolis, MN) dissolved in 250 µl of saline. Rats were sacrificed 5 h later and serum was separated from trunk blood for leptin and CORT measurements. We followed this protocol because our previous time-course study^7^ has clearly demonstrated that the HPA axis, as assessed by serum corticosterone, in normal Sprague-Dawley rats is effectively suppressed starting at 3 h, but is maximally decreased 5 h after an ip injection of leptin. PVN NE levels were also found to be decreased from as early as 30 min to 5 h post-leptin treatment^7,19^. Brains were removed and frozen immediately on dry ice and stored at −70 °C for processing as described below. In second experiment, 3-month old male DIO and DR rats (6–8 per group) were fed either SC diet or HF diet for either 1 or 6 weeks. The HF diet contained 20% protein, 35% carbohydrate, and 45% calories as fat with an energy density of 4.73 kcal/g (D12451; Research Diets Inc., New Brunswick, NJ). Food intake and body weight were measured on a weekly basis until the end of treatment. At the end of the treatment, the rats were sacrificed after 2–3 h fasting (~10am), and their brains were collected, frozen in dry ice, and stored at −70 °C. Trunk blood was collected, the serum was separated, and stored at −20 °C until RIA and ELISA analyses. Visceral fat (retroperitoneal, epidydimal, perirenal) was removed from the carcass and weighed.

### Palkovits’ microdissection

The PVN and ME, as well as noradrenergic nuclei from the brainstem (A1, ventrolateral medulla; A2, nucleus tractus solitarus; and A6, locus coeruleus) were isolated from 300 µm brain sections via micropunch tools, as described before^3,19^ using a rat brain atlas^20^. Care was taken to include all subdivisions of the nuclei from multiple serial sections. The ME was stored as such at −70 °C until analysis for CRH concentrations using ELISA. The PVN was stored in 0.1 M HClO_4_ at −70 °C and analyzed for NE concentrations using HPLC-EC. Brainstem tissue punches were stored in −70 °C for western blot analysis.

### HPLC-EC

The HPLC-EC system and the details of the mobile phase have been described previously^8,19^. Briefly, the system consisted of a Shimadzu LC-10 AT/VP pump, a phase II, 5μm ODS reverse-phase, C-18 column (Phenomenex, Torrance, CA), a glassy carbon electrode (Bioanalytical Systems, West Lafayette, IN) placed inside a Shimadzu CTO-10 AT/VP column oven at 37 °C, and a LC-4C amperometric detector (Bioanalytical Systems, West Lafayette, IN) connected to a computer with the class VP chromatopac software (Shimadzu, Columbia, MD). The mobile phase was pumped at a flow rate of 1.8 ml/min. The range of the detector was 1.0 nA full scale, and the potential of the working electrode was 0.65 V. At the time of HPLC analysis, microdissected tissue samples were thawed and homogenized in 150 μl of 0.1 M HClO_4_ using a micro-ultrasonic cell disruptor (Kontes, Vineland, NJ) and centrifuged at 10,000 × *g* for 10 min. 120 μl of the supernatant was mixed with 30 μl of the internal standard (0.05 M dihydroxybenzylamine) and 125 μl of this mixture was injected into the HPLC system. The sensitivity of the system was <1 pg. NE concentrations were expressed as pg/µg protein.

### Western blots

Noradrenergic nuclei from the brainstem were homogenized in lysis solution (Sigma Aldrich, St. Louis, MO) and protein concentrations were determined using a micro BCA assay (Pierce, Rockford, IL). Equal quantities of protein (20 µg) were loaded on to SDS-PAGE gels (NuPAGE, Invitrogen, Carlsbad, CA) and separated. Gels were blotted onto nitrocellulose membranes and the membranes were probed with pSTAT-3 antibody (1:1000; goat-polyclonal; Santa Cruz Biotechnology, Dallas, TX) and GAPDH antibody (1:2000; mouse-monoclonal; Sigma-Aldrich, St. Louis, MO). After washing, the membranes were incubated in blocking solution containing goat anti-rabbit DyLight 800 and goat anti-mouse DyLight 680 secondary antibodies (1:5000; Thermo Fisher Scientific; Waltham, MA). Bands were visualized using an Odyssey imaging system (Li-COR biosciences, Lincoln, NE).

### ELISA for leptin, CRH, and IL-1β

Serum leptin was measured in duplicate using a commercial ELISA kit (TiterZyme Kits, Assay Design, Ann Arbor, MI) according to the manufacturer’s specifications. The plates were read at 450 nm using an ELISA reader (ELx800, BioTek Instruments, Inc., Winooski, VT). The sensitivity of the kit was 46.7 pg/ml. CRH EIA kit (Phoenix Pharmaceuticals, Inc., Belmont, CA) was used to determine CRH protein in the ME. The assay had a minimum sensitivity of 0.30 ng/ml. Protein concentrations in the tissue were measured and CRH was expressed as ng/μg protein. Inter-assay variability was <5%. IL-1β was measured using a commercially available kit (Millipore, MA).

### Radioimmunoassay for CORT

A double-antibody radioimmunoassay for CORT was performed using a tracer and standards from EMD Millipore (Billerica, MA) and indigenous primary and secondary antibodies. The samples (50 μl) were assayed in duplicate as described previously^3,7^. The sensitivity of the assay was 0.2 ng/ml. The inter-assay variability was <4%.

### Circulating FFA profiles

Fatty acids in the serum were separated and quantified as described before^21^. Total lipids from serum were extracted using hexane:ethanol (1:1) solvent mix by mixing equal volumes of the serum and the solvent mix, vortexing for 10 min and centrifuging at 2,095 g for 10 min at room temperature. Fatty acids were trans-esterified and methylated using 20% methanolic hydrochloric acid for 2 h at 90 °C. Methylated extracts were analyzed using a Clarus 500 gas chromatography apparatus (Perkin Elmer, Waltham, MA) and separated on a Supelco SP-2560 column (100 m × 25 mm with a film thickness of 0.2 µm; Supelco, Bellefonte, PA). Purified methyl esters were used as standards (Nu-Chek Prep, Elysian, MN). Concentrations were expressed as mg/dl.

### Statistical analysis

In leptin experiment, changes in NE, CRH, and serum hormones were analyzed by 2-way ANOVA whereas serum lipid levels in addition to these analytes in HF experiment were analyzed by 3-way ANOVA. Serum cytokine and protein expression in the brainstem were analyzed by 2-way ANOVA. Weekly caloric intake differences in DIO & DR animals after SC or HF diet exposure were analyzed by repeated measures 2-way ANOVA. Average calorie intake and feed efficiency were analyzed by 3-way ANOVA. All ANOVAs were followed by post-hoc Fisher’s LSD test. Body weight (BW) at the end of treatment, BW gain/week during the treatment period, total white adipose tissue (visceral fat) weight, and the fat/BW ratio between DIO and DR animals within each dietary treatment were analyzed by student’s t-test.

## Results

### Exogenous leptin induces differential stress axis activity in DIO and DR rats

**Fig. 1:**
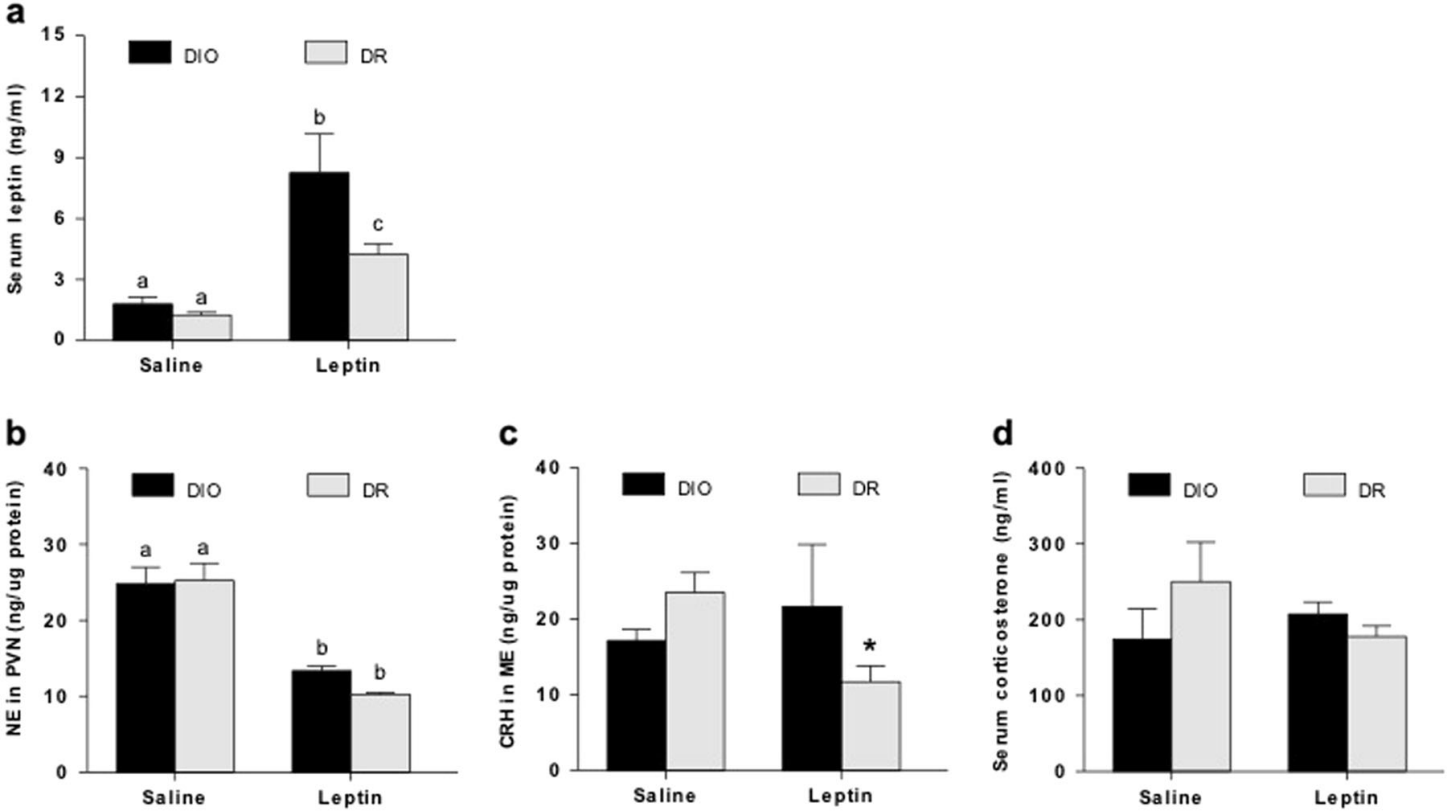
Effects of a single leptin injection on serum leptin levels and HPA axis activity in DIO and DR rats. **a**, **b** Note the marked reduction in PVN NE levels in both DIO and DR rats, indicating normal responsiveness to exogenous leptin. **c**, **d** Decrease in ME CRH or serum corticosterone in DR rats following leptin injection is not observed in DIO rats. Bars with different notations are significantly different from each other (*p* < 0.05). * indicates significant difference from the rest of the groups (*p* < 0.05). 6–8 rats/group

A single ip injection of recombinant rat leptin after overnight fasting increased serum leptin levels (ng/ml; Mean ± SE) by ~4 fold in DIO rats (8.26 ± 1.9) and produced a 2-fold increase in DR rats (4.23 ± 0.5) compared to saline-treated controls (1.8 ± 0.3 and 1.2 ± 0.1 in DIO and DR respectively; Fig. 1a). This produced a corresponding decrease in PVN NE concentrations (pg/µg protein; Mean ± SE) in both DIO (13.4 ± 0.5) and DR rats (10.4 ± 0.3) compared to controls (24.9 ± 2 and 25.3 ± 2.2 in DIO and DR rats respectively; Fig. 1b). Interestingly, while CRH levels in the ME (ng/µg protein; Mean ± SE) were reduced in leptin-treated DR rats (11.8 ± 2.1) compared to controls (23.5 ± 2.7; *p* < 0.05), no change was noted in DIO rats with leptin treatment (21.7 ± 8.2 vs. 17.2 ± 1.5 in controls; Fig. 1c). Serum CORT levels (ng/ml; Mean ± SE) also showed a trend of decrease after leptin treatment in DR rats only (Fig. 1d). Collectively, these findings demonstrate that unlike DR rats that display intact leptin action on noradrenergic neurons and NE-HPA axis circuitry, DIO rats have uncoupling between NE and HPA axis activity, suggesting inherent neuroendocrine changes in DIO rats that are independent of chronic HF diet exposure as shown in our earlier study^3^.

### Food intake and feed efficiency with 1 or 6 weeks of SC or HF diet

**Fig. 2:**
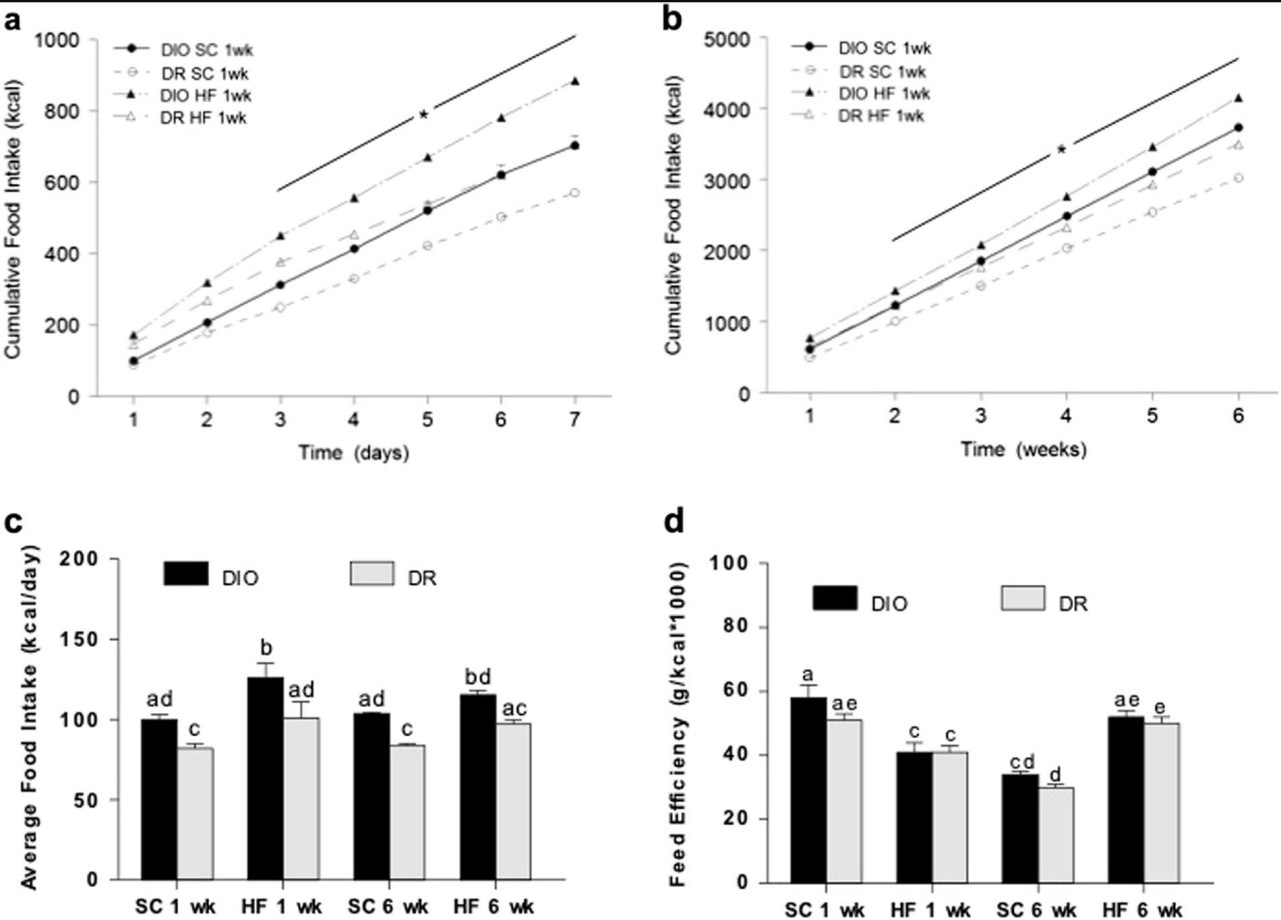
Food intake in DIO and DR rats. The effect of standard chow (SC) or high fat (HF) diet for (**a**) 1 week or (**b**) 6 weeks on cumulative food intake. Food intake in HF-fed animals were significantly different from chow-fed animals. **c** Average food intake/day in the different treatment groups. **d** Average feed efficiency. * indicates significant difference between DIO and DR group on the same diet. Bars with different notations are significantly different from each other (*p* < 0.05). 6–8 rats/group

In the 1 week groups, cumulative chow intake (kcal) was higher in DIO compared to DR rats starting on the third day and remained higher at the end of the week (Fig. 2a). Cumulative HF intake was greater in DIO rats as early as the second day that continued throughout. A similar trend was observed for rats exposed to 6 weeks of chow or HF diet (Fig. 2b). Cumulative food intake was higher in both chow and HF-fed DIO rats compared to their DR counterparts. This is likely attributed to an increased daily average food intake in DIO vs. DR rats, and between HF vs. SC feeding (Fig. 2c). In agreement with earlier findings by Levin and colleagues^22^, there were no significant differences in feed efficiency between DIO and DR rats across treatments (Fig. 2d).

### Body weight and adiposity in DIO and DR rats after 1 or 6 weeks of SC or HF diet

BW gain is expressed per week to eliminate confusion between different duration of treatments. Even with chow diet, DIO rats gained significantly more body weight per week (mean ± SE; g; 21.1 ± 0.6) than DR rats (15.8 ± 0.7; *p* < 0.001). One week of HF exposure did not produce any significant change in the BW gain between the two phenotypes (*p* < 0.08). However, prolonging the duration of HF diet to 6 weeks significantly increased BW gain in DIO (35.9 ± 1.3) compared to DR animals (29.7 ± 1.2; *p* < 0.05; see Supplementary information Table S1).

### NE-HPA axis responsiveness to leptin is impaired after acute HF feeding and is further exacerbated after prolonged HF feeding in DIO rats

**Fig. 3:**
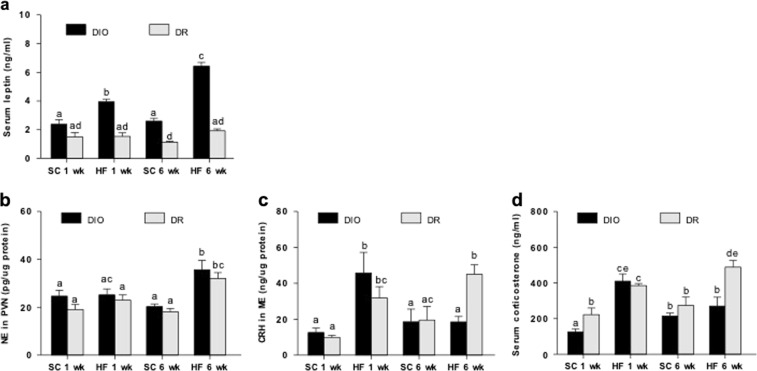
Effects of different duration of HF diet exposure on serum leptin levels and HPA axis activity in DIO and DR rats. **a**, **b** Note that PVN NE levels in DIO rats are elevated after 6 weeks of HF in spite of higher leptin levels. **c**, **d** DR rats have an activated stress axis in response to HF diet exposure, while DIO rats do not. Bars with different notations are significantly different from each other (*p* < 0.05). 6–8 rats/group

While serum leptin levels (ng/ml; Mean ± SE) did not change in DIO rats under SC diet (2.4 ± .3 vs. 2.6 ± 0.2 at 1 and 6 weeks; Fig. 3a), HF feeding for 1 week significantly increased (3.9 ± 0.2) and 6 weeks further elevated serum leptin (6.4 ± 0.3; *p* < 0.05). However, leptin levels were not altered in DR rats and this was independent of treatment duration. Next, we assessed HPA axis circuitry in these animals. In spite of no change in PVN NE between DIO and DR rats across treatments (Fig. 3b), 6 weeks of HF diet increased NE levels in both DIO and DR rats. HF feeding for 1 week, but not 6 weeks, markedly increased ME CRH (ng/µg protein; Mean ± SE; 45.8 ± 11.5 1wk vs. 18.5 ± 3.0 6wks) compared to that under SC diet (18.6 ± 7.1; *p* < 0.05) in DIO rats. (Fig. 3c). On the contrary, CRH levels in DR rats increased after 1 week of HF diet (31.9 ± 6.3) and continued to increase after 6 weeks (45.1 ± 5.2) compared to those under SC diet (19.5 ± 7.5; *p* < 0.05). Serum CORT levels (ng/ml; Mean ± SE) elevated similarly in both DIO and DR rats after 1 week of HF diet compared to those in SC-fed animals (Fig. 3d). However, after 6 weeks of HF feeding, CORT continued to increase only in DR rats (488.7 ± 36.6 vs. 384.5 ± 12.6 1wk) and not in DIO rats. It was in fact significantly lowered in DIO rats (270.5 ± 49.2 6wks vs. 409.7 ± 41.3 1wk; *p* < 0.05). These findings suggest that acute HF feeding impairs leptin action to suppress noradrenergic activity in DIO rats, and prolonged HF feeding further worsens the coupling of NE-HPA axis circuitry. Results from NE, CRH and CORT in response to exogenous leptin or HF treatment in DIO and DR rats are summarized in Supplementary information Table S2.

### pSTAT-3 expression is reduced in brainstem noradrenergic neurons in DIO rats

**Fig. 4:**
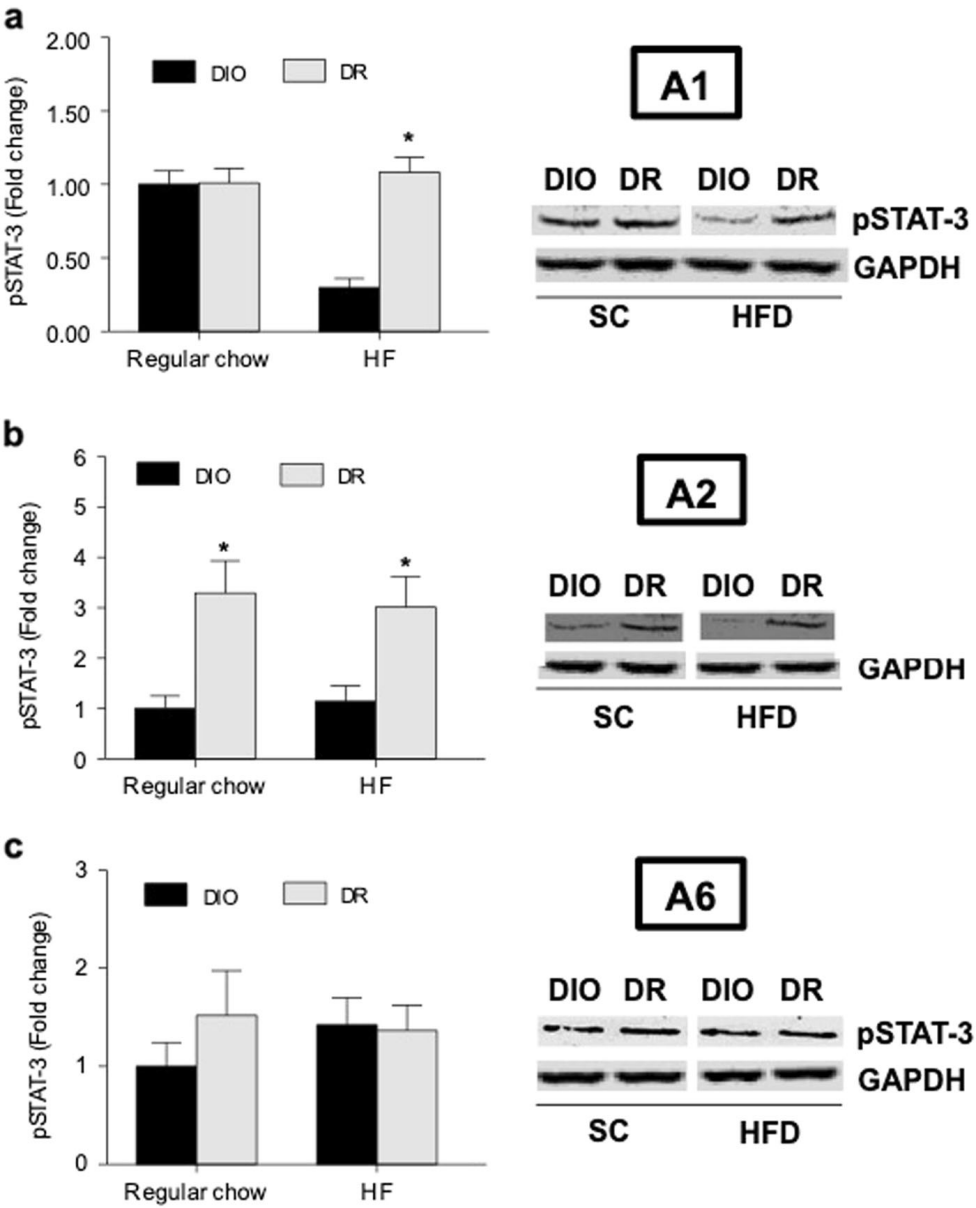
Phosphorylation of STAT-3 in brainstem NE neurons after chow or HF diet exposure for 6 weeks. **a** Results from A1 noradrenergic nucleus; **b** A2 noradrenergic nucleus and **c** A6 noradrenergic nucleus. Data are normalized to GAPDH and expressed as a fold change from regular chow-fed DIO group. * indicates significant difference from the corresponding DIO group (*p* < 0.05). 6–8 rats/group

To determine possible reasons for the inability of endogenous leptin to suppress PVN NE, we measured pSTAT-3 protein levels in specific brainstem areas to assess leptin signaling. These areas contain mostly noradrenergic neurons. The expression of pSTAT-3 (Fold change; Mean ± SE) was markedly reduced in A1 region of DIO rats (0.29 ± 0.06) compared to DR rats (1.08 ± 0.1; *p* < 0.05) after 6 weeks of HF diet (Fig. 4a). This nucleus provides the majority of noradrenergic innervation to the PVN where CRH neurons are localized^23^. In contrast, a significant reduction in pSTAT-3 expression was observed in A2 region of DIO rats irrespective of diet (Fig. 4b). There was no change in A6 region (Fig. 4c).

### Circulating FFAs and IL-1β are increased in DIO rats after HF feeding

**Fig. 5:**
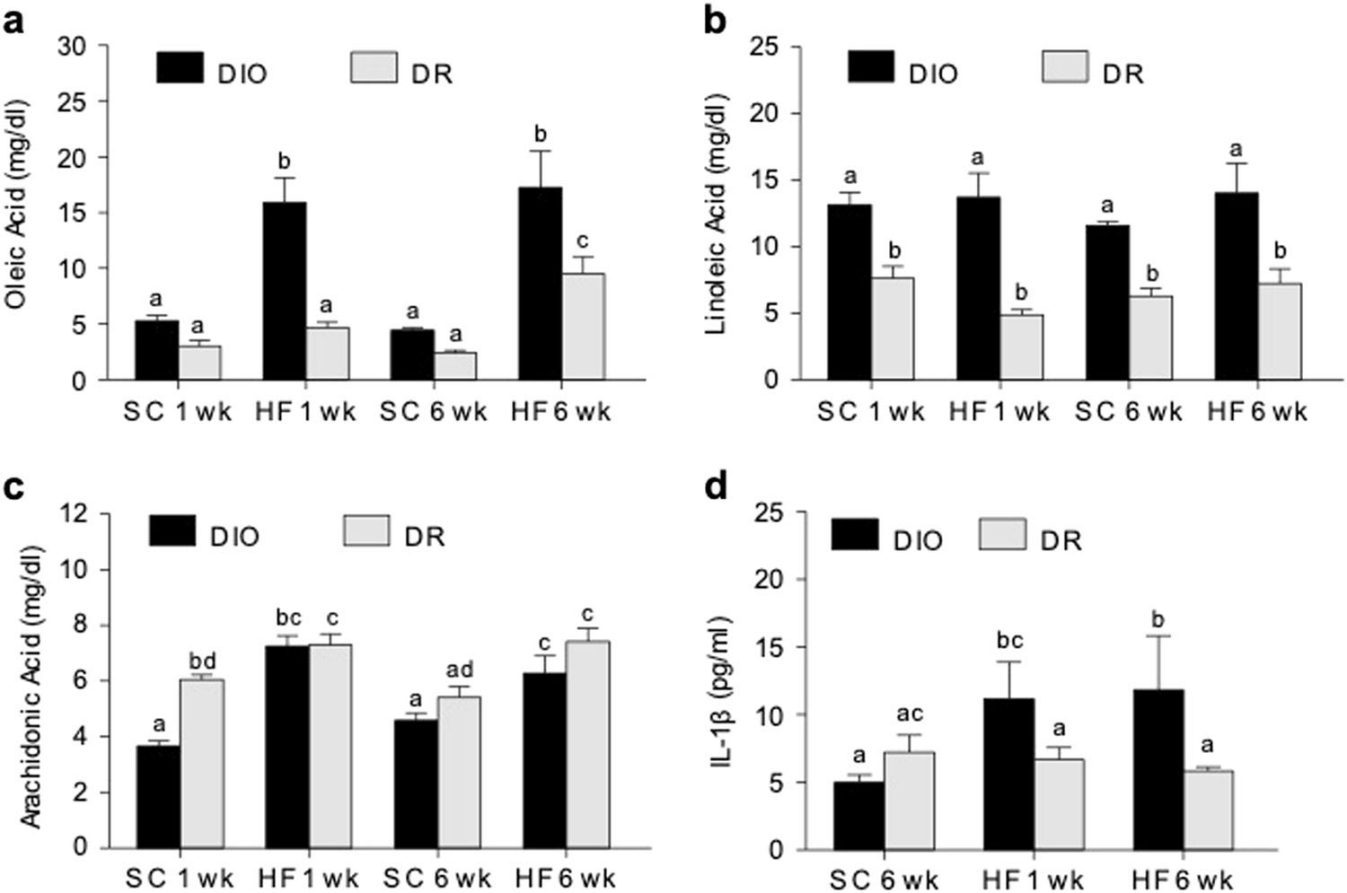
Serum free fatty acids and IL-1β levels. **a** Oleic acid, **b** Linoleic acid, and **c** Arachidonic acid levels were measured by GC-MS. **d** IL-1β levels were measured in SC-fed (6 weeks) rats compared to HF-fed DIO and DR rats. SC 1wk group was not included because IL-1β was below detection level. Bars with different notations are significantly different from each other (*p* < 0.05). Note that DIO rats have higher levels of serum IL-1β after 1 or 6 weeks of HF diet exposure. 6–8 rats/group

Serum oleic acid levels (mg/dl; Mean ± SE) were significantly increased in DIO rats after HF diet, but only after 1 week of HF diet for DR rats (Fig. 5a). Linoleic acid levels were markedly increased in DIO rats compared to DR rats irrespective of diet and duration (Fig. 5b). Arachidonic acid levels similarly increased in DIO and DR rats under HF diet (Fig. 5c). Serum IL-1β (pg/ml; Mean ± SE; Fig. 5d) increased significantly only in DIO rats under HF diet.

## Discussion

While the metabolic phenotype of polygenically bred DIO and DR rats has been extensively studied previously by Levin and colleagues^22,24,25^, our knowledge on potential neuroendocrine changes in DIO rats is somewhat limited to alternations in amygdalar CRH/GR gene expression and circulating glucocorticoid levels^26–28^. The underlying mechanism of altered HPA axis activity is also not well understood. We have previously identified leptin as an important regulator of HPA axis through suppressing α-adrenergic signaling^7^, and our comprehensive analysis of leptin and NE-HPA axis circuitry – NE in the PVN, CRH in the ME, serum corticosterone – demonstrated an uncoupling within the system in DIO but not DR rats chronically exposed to HF diet^3^. It was not clear, however, if this phenomenon is due to inherent genetic differences between them or is a result of HF feeding-related changes. We tested this by giving a single dose of systemic leptin injection in DIO and DR rats under chow diet, and placed them on a HF diet for either 1 or 6 weeks to observe HF-related neuroendocrine changes as a function of time. Our current study extends previous research and shows novel findings that (1) increased noradrenergic tone in DIO rats may be due to leptin resistance in the brainstem and (2) dysregulation of the HPA axis and impaired leptin action on PVN NE under HF diet worsens in a time-dependent manner.

A single leptin injection significantly increased circulating leptin levels in both DIO and DR rats, albeit higher levels found in DIO rats. This is in agreement with studies showing reduced clearance rate for leptin in obese vs. lean subjects and increased clearance following an acute fasting^29,30^. This resulted in a marked decrease in PVN NE in both groups, a response very similar to what was observed in lean rats from previous studies^7,19,31,32^. The result also indicates that the brainstem noradrenergic system in DIO and DR rats remain sensitive to elevation of circulating leptin. Unlike NE, ME CRH levels and serum CORT decreased only in DR and remained unchanged in DIO rats (Supplementary Table S2 online), suggesting possible leptin/NE insensitivity in CRH neurons of DIO rats. While CRH is also expressed in other brain regions such as bed nucleus stria terminalis and amygdala^33^, the only source of CRH released into the ME before reaching the anterior pituitary is CRH neurons in the PVN. The lack of CORT response to leptin in DIO rats is unclear, but it is likely that other circulating factors such as pro-inflammatory cytokines and FFAs as discussed below counter the suppressive effects of leptin on CORT. The HPA responses in DR rats are very similar to what we have shown previously with leptin treatment in lean Sprague-Dawley rats^7,19^, suggesting that they have normal stress axis function.

Regardless of diets, DIO rats consumed more calories compared to DR rats, and their increase in fat mass likely accounts for the higher serum leptin seen especially under HF diet. On the other hand, we found a modest increase in fat mass in DR animals after 6 weeks of HF diet without affecting leptin levels. These results are in agreement with a previous finding showing that HF exposure increased serum leptin in DIO rats but not in DR rats^22^. When we examined the effects of HF diet on PVN NE, we found that NE levels were significantly higher in both genotypes after 6 weeks (Supplementary Table S2 online). Importantly, the inability of DIO rats to suppress PVN NE in spite of elevated leptin levels indicates impaired leptin action. Conversely, NE levels may increase in response to other HF-related circulating factors as discussed below. ME CRH and serum CORT increased in both groups after 1 week of HF diet, but only in DR rats after 6 weeks of HF diet. Collectively, the increase of PVN NE, CRH ME, and serum CORT in DR rats suggests that they perceive HF diet as a stressor. On the other hand, the continuous rise of PVN NE in the face of hyperleptinemia, and the initial, parallel rise in CRH and CORT and fall later in HF-fed DIO rats indicates dysregulated stress axis that becomes exacerbated as a function of time. Whether the reduced HPA axis activity seen in chronic HF-fed DIO rats leads to lower EE and differential substrate utilization that may be responsible for development of obesity needs further investigation.

Since functional leptin receptor (ObRb) is present in all A1, A2, and A6 regions in the brainstem^34–37^, we examined the possibility that leptin insensitivity localized to noradrenergic neurons in these areas contributes to higher NE levels in the PVN of DIO rats. Indeed, our results indicate that phosphorylation of STAT-3, a critical transcription factor for leptin signaling, decreases significantly in A1 region after HF feeding, and it is perhaps constitutively expressed at a low level in A2 region in DIO rats. Although we did not inject exogenous leptin to determine leptin signaling, we were able to significantly raise endogenous leptin in DIO rats by feeding a HF diet. Diet-induced obesity is associated with a low-grade inflammation with increased circulating pro-inflammatory cytokines^38,39^. As STAT-3 can be phosphorylated not only by leptin but also by these cytokines including TNF-α, IL-6, and IL-1β^40^, pSTAT-3 may be expected to increase by elevated IL-1β we observed in HF-fed DIO rats. However, our finding that it rather decreased strongly suggests that lower pSTAT-3 in the brainstem of DIO rats primarily reflects leptin resistance and not cytokine signaling. Whether or not acute leptin-stimulated pSTAT-3 is lower after HF feeding in DIO rats would need to be tested.

Reduced leptin signaling in the brainstem of DIO rats may also be due to lack of leptin availability in the brain. Studies show that leptin’s ability to cross the blood brain barrier can be impaired by HF feeding or circulating FFAs or triglycerides^12,14,41^. In this study, we found HF- and time-dependent increase in oleic acid and arachidonic acid in DIO rats. It is notable that arachidonic acid is also able to induce leptin resistance in the brain^42^. Serum linoleic acid was markedly elevated in DIO rats regardless of treatment, therefore it is difficult to assign a specific role of this FFA. Furthermore, most mono or polyunsaturated FAs such as above are also known to directly activate HPA axis^13,17,18^. Thus, these FFAs may participate in stimulating noradrenergic tone and/or HPA activity as seen in DIO rats via multiple mechanisms. Besides circulating FFAs, pro-inflammatory cytokines could also contribute to HPA axis dysfunction. In the present study, serum IL-1β levels were markedly increased in DIO animals under HF diet that could have stimulatory effects on the HPA axis^15,16^. We speculate that HF-induced increase in IL-1β at least partly overrides leptin’s suppressive effects on the HPA axis leading to its dysfunction in these rats.

Beyond its role in the control of HPA axis, leptin is better known for its action in the long-term regulation of body weight and food intake^43,44^. Intracerebroventricular (icv) CRH infusion lowers food intake in rats^45,46^ whereas CRH receptor antagonist attenuates leptin-induced reduction of food intake^47^, suggesting that CRH is critical for leptin-mediated appetite suppression. Interestingly, DIO rats develop leptin resistance in the ARC before exposed to HF diet^41^, leading us to predict that CRH release from the PVN in HF-fed DIO rats will be lower compared to that in chow-fed DIO rats. On the contrary, our results demonstrate significantly increased ME CRH in 1 week HF-fed DIO rats compared their chow-fed counterparts, suggesting that this is most likely due to leptin resistance in the brainstem and not in the ARC. The potential contribution of intact leptin signaling in the ARC of DR rats on the overall ME CRH increase after HF feeding warrants further investigation.

**Fig. 6:**
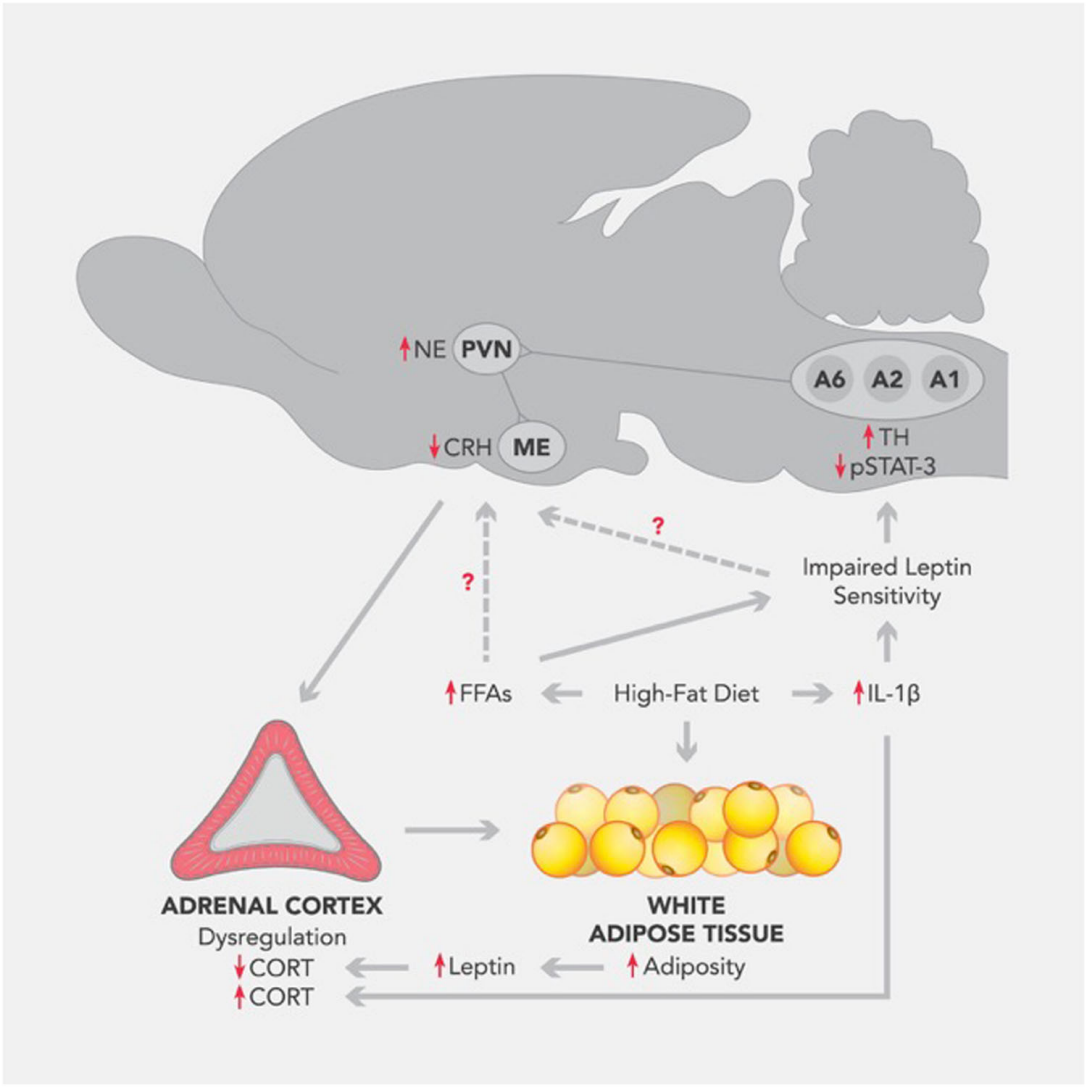
Working model for dysfunctional HPA axis activity in DIO rats. Unlike DR rats that display appropriate NE-HPA responses to an exogenous leptin, DIO rats already show an uncoupling of the NE-HPA axis circuitry. Once exposed to HF diet, DIO rats exhibit worsened leptin signaling in the brainstem in a time-dependent manner that leads to increased noradrenergic input to CRH neurons in the PVN, however uncoupling to HPA axis ensues. We speculate that the early leptin resistance in NE neurons and exacerbated NE-HPA axis uncoupling likely lead to the development of obesity in the selectively bred DIO rats. Whether these neuroendocrine defects are attributed to high circulating FFAs or leptin resistance in DIO rats needs further investigation

In summary, the results from this study indicate that the noradrenergic system in the brainstem remains responsive to exogenous leptin in DIO and DR animals. However, when they are placed on a HF diet, there is a time-dependent impairment of leptin action to suppress noradrenergic tone in the PVN of DIO rats that is most likely due to reduced pSTAT-3 expression in specific noradrenergic nuclei. DIO rats also appear to develop HPA axis dysfunction with prolonged HF diet exposure. Possible mediators of these phenomena include higher serum FFAs and pro-inflammatory cytokines such as IL-1β. These may in turn override the NE-HPA suppressive effects of leptin promoting development of obesity in this polygenically obese model (Fig. 6).

## Supplementary information

Supplementary Table S1 and Table S2
