# Supplementary material for: Responsiveness of hypothalamo-pituitary-adrenal axis to leptin is impaired in diet-induced obese rats
Source: Nutr Diabetes. 2019 Mar 18;9:10. doi: 10.1038/s41387-019-0076-y (PMC6423225; doi:10.1038/s41387-019-0076-y)
Supplement: Supplementary file 1 — Supplementary Table S1 and Table S2 [file 41387_2019_76_MOESM1_ESM.docx]

|  | | | |  |
| --- | --- | --- | --- | --- |
| Treatment | Parameters | *DIO* | *DR* | |
|  |  |  |  | |
|  | Final body weight (BW; g) | 365.3±4.6* | 281.1±4.8 | |
| Chow 1 wk | BW gain/week (g) | 40.4±2.3* | 29.2±1.5 | |
|  | Total adipose tissue weight (AW; g) | 8.7±1.1* | 3.7±0.2 | |
|  | AW to BW ratio (%) | 2.4±0.3* | 1.3±0.2 | |
|  |  |  |  | |
|  | Final body weight (BW; g) | 395.4±7.7* | 310.1±5.0 | |
| Chow 6 wks | BW gain/week (g) | 21.1±0.6* | 15.8±0.7 | |
|  | Total adipose tissue weight (AW; g) | 13.5±1.1* | 5.2±0.2 | |
|  | AW to BW ratio (%) | 3.4±0.2* | 1.7±0.1 | |
|  |  |  |  | |
|  | Final body weight (BW; g) | 403.0±2.7* | 315.6±3.6 | |
| HF 1 wk | BW gain/week (g) | 36.0±1.6; p = 0.08 | 28.9±2.6 | |
|  | Total adipose tissue weight (AW; g) | 10.5±0.6* | 6.4±0.3 | |
|  | AW to BW ratio (%) | 2.6±0.2* | 2.0±0.1 | |
|  |  |  |  | |
|  | Final body weight (BW; g) | 525.9±13.3* | 421.0±9.1 | |
| HF 6 wks | BW gain/week (g) | 35.9±1.3* | 29.7±1.2 | |
|  | Total adipose tissue weight (AW; g) | 22.0±1.6* | 10.2±0.7 | |
|  | AW to BW ratio (%) | 4.2±0.3* | 2.4±0.2 | |

**Supplementary Table S1.** *Comparison of body weight and white adipose tissue deposition between DIO and DR rats placed on chow or HF diet for either 1 or 6 weeks.* * p<0.05 compared to DR rats based on student’s t-test.

| **DIO** | **Treatment** | **Leptin** | **NE** | **CRH** | **Corticosterone** |
| --- | --- | --- | --- | --- | --- |
|  | **Leptin** | ↑↑ | ↓ | ̶ | ̶ |
|  | **HF 1wk** | ↑ | ↑ | ↑ | ↑ |
|  | **HF 6wk** | ↑↑ | ↑↑ | ̶ | ̶ |
|  |  |  |  |  |  |
| **DR** | **Treatment** | **Leptin** | **NE** | **CRH** | **Corticosterone** |
|  | **Leptin** | ↑ | ↓ | ↓ | ↓ |
|  | **HF 1wk** | ̶ | ↑ | ↑ | ↑ |
|  | **HF 6wk** | ̶ | ↑↑ | ↑↑ | ↑↑ |

**Supplementary Table S2.** *Summary of HPA responses in DIO and DR rats after a single injection of leptin or either 1 or 6 weeks of HF diet exposure compared to their saline or chow-fed controls. – indicates no change compared to baseline.*
